# Advances in RNA-Based Therapeutics: Challenges and Innovations in RNA Delivery Systems

**DOI:** 10.3390/cimb47010022

**Published:** 2024-12-31

**Authors:** Yuxuan Liu, Yaohui Ou, Linlin Hou

**Affiliations:** School of Medicine, Shenzhen Campus of Sun Yat-sen University, Shenzhen 518107, China; liuyx355@mail2.sysu.edu.cn (Y.L.); ouyh36@mail2.sysu.edu.cn (Y.O.)

**Keywords:** RNA-based therapeutics, RNA delivery systems, viral vectors, virus-like particles (VLPs), lipid nanoparticles (LNPs), extracellular vesicles (EVs), nanomaterial-based delivery, immune response, targeted therapy

## Abstract

Nucleic acids, as carriers of genetic information, have found wide applications in both medical and research fields, including gene editing, disease diagnostics, and drug development. Among various types of nucleic acids, RNA offers greater versatility compared to DNA due to its single-stranded structure, ability to directly encode proteins, and high modifiability for targeted therapeutic and regulatory applications. Despite its promising potential in biomedicine, RNA-based medicine still faces several challenges. Notably, one of the most significant technical hurdles is achieving efficient and targeted RNA delivery while minimizing immune responses. Various strategies have been developed for RNA delivery, including viral vectors, virus-like particles (VLPs), lipid nanoparticles (LNPs), and extracellular vesicles (EVs). In this review, we explore the applications of these delivery methods, highlight their advantages and limitations, and discuss recent research advancements, providing insights for the future of RNA-based therapeutics.

## 1. Introduction

Nucleic acid and genetic drugs are increasingly being applied in the biomedical field, covering gene therapy, vaccine development, and disease diagnostics [1,2]. RNA-based therapies include various approaches to treat different medical conditions. Messenger RNA (mRNA) therapy delivers synthetic mRNA to produce proteins and induce immune responses, with applications in vaccines, cancer immunotherapy, and genetic disorders [3]. Small interfering RNA (siRNA) silences specific genes, while antisense oligonucleotides (ASOs) and microRNAs (miRNAs) modify gene expression, offering potential treatments for diseases like viral infections, cancer, and genetic disorders [4,5,6]. Additionally, ribozyme-based therapy, which targets disease-causing RNAs, is in early development for genetic disorders [7].

RNA-based drugs have gained significant attention due to their ability to regulate gene expression post-transcriptionally, particularly through RNA interference (RNAi) mechanisms involving siRNAs and miRNAs [8]. These molecules can precisely target and degrade mRNA or inhibit its translation, enabling control over protein production without altering the genome—an advantage over DNA-based therapies [9]. RNA therapies also circumvent limitations of DNA approaches, such as the need to enter the nucleus, since RNA functions primarily in the cytoplasm. This makes RNA delivery simpler and reduces the risk of genomic integration. mRNA vaccines, for example, leverage the cell’s translational machinery to produce proteins [10,11]. Furthermore, RNA can be chemically modified to enhance stability and targeting, making it adaptable for a wide range of therapeutic applications. The success of siRNA drugs, such as Patisiran (ONPATTRO™), Bevasiranib, AGN211745 [4], and mRNA-based vaccines in combating the COVID-19 pandemic, underscores RNA’s critical role in pharmaceutical applications and heralds a new era in RNA-based medicine [12].

Despite their potential, the effective application of RNA molecules faces numerous challenges, with stable and efficient delivery into target cells being the most critical [13,14]. In recent years, significant progress has been made in RNA delivery technologies, incorporating a variety of strategies and carriers that have greatly improved delivery efficiency and safety [15]. In this review, we discuss four primary RNA delivery systems: viral vectors, virus-like particles (VLPs), lipid nanoparticles (LNPs), and extracellular vesicles (EVs) (Table 1), while also exploring other nanomaterial-based platforms. We highlight their respective advantages and limitations, summarize recent research advancements, and explore future directions and applications in RNA therapeutics (Table 2).

## 2. Viral Delivery

Viral vectors are among the most extensively studied RNA delivery systems, with adeno-associated virus (AAV), adenovirus (AdV), herpes simplex virus (HSV), retrovirus, and lentivirus being the most commonly used. These vectors exploit the natural infection mechanisms of viruses to efficiently introduce RNA into cells, facilitating effective gene transfer [47,48]. Viral vectors are capable of expressing RNA efficiently and persistently, thus holding significant potential in gene therapy and vaccine development. However, the immunogenicity of viral vectors and the associated risks of genomic integration limit their clinical applicability (Table 3) [49,50]. Retroviral and lentiviral vectors, for instance, have seen declining use due to the high risk of insertional mutagenesis [51]. While AdV and HSV deliver nucleic acids episomally, their use is often associated with severe inflammation, immune responses, and challenges in genetic manipulation. In contrast, AAV has emerged as a preferred vector in research and clinical settings, owing to its low immunogenicity and capacity for stable gene expression, particularly in gene therapy and RNA delivery applications [51].

AAV is a small, non-enveloped virus capable of packaging single-stranded DNA (ssDNA) [52]. It has been extensively engineered for DNA delivery by replacing all viral protein-coding sequences with a therapeutic gene expression cassette flanked by two essential packaging signals, the inverted terminal repeats (ITRs) [48]. Unlike retrovirus-derived virus-like particles (VLPs), where virus assembly and genome encapsidation occur simultaneously, the AAV genome is synthesized separately and then loaded into a pre-assembled capsid. This process occurs in a 3′ to 5′ direction, driven by the viral DNA helicase/ATPase activity of Rep proteins (Rep78, Rep68, Rep52, and Rep40) [53,54]. The non-structural proteins Rep78 and Rep68 play a dual role, not only acting as motors but also bridging the ssDNA genome to the pre-assembled capsid during packaging [52,54]. Building on these mechanisms, Bai et al. replaced the ITRs with RNA packaging signals (RPSs), allowing Rep78/68 proteins to bind RPS-harboring mRNAs and transform AAV into an RNA-packaging virus. This innovation led to the development of mRNA-carrying AAVs (RAAVs), which demonstrated high mRNA selectivity and minimal residual DNA packaging (~0.005%). In central nervous system (CNS) oncology, gene therapy has faced limited clinical efficacy due to the blood-brain barrier (BBB), a major anatomical and functional obstacle, leaving surgery as a primary treatment option [55]. Notably, RAAVs have shown the ability to mediate efficient mRNA transfer into target cells and tissues, enabling transient expression of functional proteins. The intravenous administration of RAAVs has allowed them to cross the BBB, successfully deliver mRNA into the mouse hippocampus, and achieve widespread infection of the entire brain. These properties make RAAVs a powerful tool for both basic neuroscience research and therapeutic applications [16].

In addition to mRNA delivery, AAVs have been utilized to package and deliver circular RNAs (circRNAs). Alzheimer’s disease (AD), a neurodegenerative disorder characterized by memory decline and cognitive dysfunction, is driven by amyloid-beta (Aβ) plaque deposition and neuroinflammation, with the latter playing a central etiopathogenic role [1,2,3,4,5]. Neuroinflammation exacerbates Aβ and Tau pathologies, partly through double-stranded RNA (dsRNA)-activated protein kinase R (PKR), whose overactivation contributes to synaptic and cognitive deficits [6]. PKR depletion has shown therapeutic promise in AD models [7,8,9,10]. Feng et al. developed circular RNAs with short-imperfect duplex regions (ds-cRNAs) containing ITRs for AAV packaging, designed to selectively inhibit PKR overactivation. Using AAV2/9 vectors, they achieved precise delivery of these therapeutic ds-cRNAs to neurons and microglia in AD mouse models. The localized PKR inhibition reduced neuroinflammation and Aβ plaque deposition, resulting in significant improvements in spatial learning and memory, with minimal toxicity. This study highlights the potential of AAV as a carrier for circRNA-based therapeutics in treating neurodegenerative diseases like AD [17].

A similar AAV-based approach has also been explored for amyotrophic lateral sclerosis (ALS), a condition characterized by motor neuron death caused by nuclear depletion and cytoplasmic aggregation of the splice factor TDP-43. Pathological TDP-43 is associated with stress granules (SGs), and reducing the SG-associated protein Ataxin-2 (Atxn2) using ASOs has been shown to prolong survival in TAR4/4 sporadic ALS mouse models—a strategy now under clinical investigation. Building on this, Amado et al. designed miRNAs targeting *Atxn2*, flanked by AAV2 145-bp ITR sequences, and packaged them into a peptide-modified AAV9 (PM-AAV9) vector engineered for enhanced central nervous system (CNS) targeting [18,56]. A single intracerebroventricular (ICV) injection of this construct early in life achieved sustained knockdown of *Atxn2* throughout the mouse CNS, demonstrating its therapeutic potential in ALS models [18].

## 3. Virus-like Particle Delivery

Virus-like particles (VLPs) are nanoscale structures composed of viral proteins but devoid of viral genetic material, rendering them non-infectious. These VLPs can be produced in a variety of systems, including mammals, plants, insects, and bacteria, and serve as versatile carriers for delivering bio- and nanomaterials, such as drugs, vaccines, quantum dots, and imaging agents, due to the cavity within their structure [57]. Additionally, VLPs are capable of efficiently encapsulating and protecting RNA, allowing for targeted delivery with high precision and minimal risk of genome integration. Recent advancements have focused on optimizing VLP design to improve delivery efficiency and cell-specific targeting, positioning them as a safer alternative to traditional viral vectors for therapeutic applications [58,59].

Various viral structural proteins have been utilized to generate VLPs, among which those derived from the human immunodeficiency virus (HIV) are the most widely used. Zhang et al. utilized HIV-derived VLPs to develop a protective mRNA vaccine aimed at controlling the HIV/AIDS pandemic. This mRNA vaccine, which co-expressed membrane-anchored HIV-1 envelope (Env) and simian immunodeficiency virus (SIV) Gag proteins to generate VLPs, induced antibodies capable of broad neutralization and significantly reduced infection risk in rhesus macaques. Immunization with a co-formulated Env and Gag mRNA combination was more effective than immunization with *Env* mRNA alone in generating neutralizing antibodies. In the study, macaques were primed with a transmitted-founder clade-B *Env* mRNA (lacking the N276 glycan), followed by multiple booster immunizations with glycan-repaired autologous Env and bivalent heterologous Envs (clades A and C). This immunization regimen was highly immunogenic, stimulating neutralizing antibodies against prevalent HIV-1 strains (tier-2) and robust anti-Env CD4+ T-cell responses. Vaccinated animals showed a 79% reduction in per-exposure risk following repeated low-dose mucosal challenges with heterologous tier-2 simian–human immunodeficiency virus (SHIV AD8), highlighting the potential of the multiclade Env-Gag VLP mRNA platform as a promising strategy for the development of an HIV-1 vaccine [19].

Building on a similar approach, Bjorkman’s team utilized HIV structural proteins Gag and Env to develop an enhanced mRNA vaccine for SARS-CoV-2. They engineered self-assembling enveloped VLPs by incorporating the endosomal sorting complex required for transport (ESCRT) and the ALG-2-interacting protein X (ALIX) binding region into the cytoplasmic tail of the SARS-CoV-2 spike protein. This modification successfully recruited ESCRT proteins to produce spike-enriched enveloped VLPs. Preclinical studies showed that mRNA vaccines encoding this modified spike protein elicited a robust immune response, particularly against variants such as Omicron, with enhanced CD8+ T cell activity and improved antibody neutralization [20]. This hybrid approach, combining mRNA technology with VLPs, holds significant promise for the development of vaccines against COVID-19 and other pathogens.

**Figure 1:**
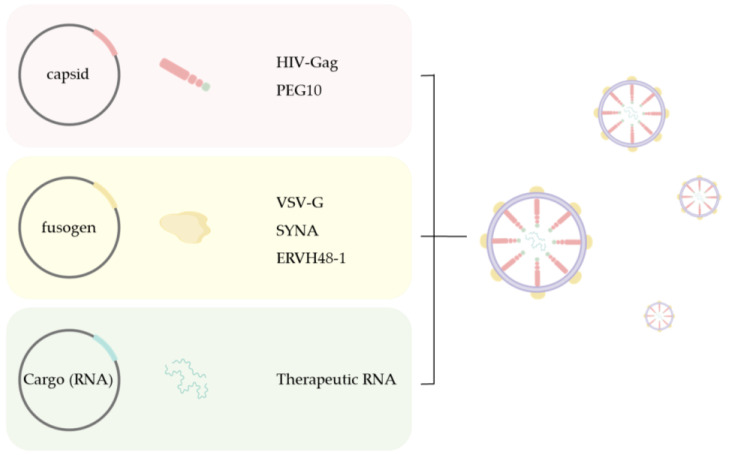
Schematic showing the components of SEND. SEND is a delivery platform that integrates an endogenous Gag homolog, a fusogen (glycoprotein), and cargo mRNA, allowing customization for specific applications.

Mammalian genomes contain domesticated genes from long terminal repeat (LTR) retroelements that have integrated throughout evolution [60,61,62,63,64]. Among these genes are homologs of the capsid protein Gag, from LTR retrotransposons and retroviruses. Zhang and his colleagues identified several mammalian Gag homologs that form VLPs, particularly PEG10, which preferentially binds to and facilitates the vesicular secretion of its own mRNA. They developed selective endogenous eNcapsidation for cellular delivery (SEND) by engineering mouse and human PEG10 to package, secrete, and deliver specific RNAs (Figure 1). In this platform, mRNA consisting of both the 5′ and 3′ untranslated regions (UTRs) of PEG10 flanking a gene of interest was efficiently packaged in recipient cells expressing endogenous fusogen, such as SYNA. The PEG10 VLPs are pseudo-typed and secreted within extracellular vesicles that mediate target RNA transfer into recipient cells. Due to its use of endogenous proteins, SEND may exhibit reduced immunogenicity compared to currently available viral vectors [21]. In addition, PEG10 is highly expressed in the thymic epithelium of the developing human thymus, playing a crucial role in T cell tolerance induction [65]. As a fully endogenous system, SEND represents a promising approach for developing minimally immunogenic delivery platforms for nucleic acid therapies [66].

## 4. Lipid Nanoparticle Delivery

Liposomes, composed of phospholipid bilayers, are amphiphilic structures featuring a polar head group, a hydrophobic tail region, and a linker domain. Their ability to encapsulate and protect nucleic acids, combined with their biocompatibility and customizability, has made them indispensable in drug delivery systems [67,68]. Surface modifications further enable liposomes to achieve targeted delivery, enhance efficiency, and provide multifunctionality with minimal toxicity—key factors for effective RNA delivery [69].

Lipid nanoparticles (LNPs) are liposome-like structures, which are ideal for encapsulating and delivering various nucleic acids. In clinical LNPs, the surface charge is typically near-neutral under physiological conditions [70]. Interestingly, a recent study revealed that using an acidic buffer could impart positive charges to LNPs, improving their stability during nebulization and enabling efficient mRNA delivery to the lungs without significant toxicity [22]. These results indicate that the lungs may demonstrate better tolerance to positively charged nanoparticles, which frequently raise toxicity concerns following systemic injections in clinical studies. Building on these findings, Liu et al. developed a charge-assisted stabilization (CAS) strategy to enhance LNP colloidal stability via electrostatic repulsion. By incorporating peptide-lipid conjugates, the CAS-LNPs maintained structural integrity during nebulization and efficiently delivered mRNA to the lungs of mice, dogs, and pigs. These CAS-LNPs preferentially targeted lung dendritic cells, eliciting robust mucosal and systemic immune responses. Notably, they demonstrated potential as vaccines against the SARS-CoV-2 Omicron variant and as cancer vaccines for preventing lung metastases. These innovations not only address the instability and aggregation of LNPs during nebulization but also establish critical design principles for inhalable mRNA vaccines and therapeutics, opening new possibilities for treating pulmonary diseases and developing mucosal vaccines [23].

Li et al. combined machine learning with combinatorial chemistry to optimize ionizable lipids for mRNA delivery [24]. They developed a four-component reaction (4CR) platform that produced a library of 584 ionizable lipids, using machine learning to identify the most effective candidates. This approach demonstrated enhanced mRNA delivery, particularly in muscle and immune cells, while reducing off-target effects. Norimatsu et al. explored triphenylphosphonium (TPP)-modified cationic polymers to stabilize polyion complexes for mRNA delivery [71]. Their TPP-modified carriers significantly improved mRNA packaging and delivery, particularly in solid tumors, where they enhanced protein expression. Similarly, Han’s team introduced amidine-incorporated degradable lipids (AID-lipids), created through a fast one-pot multi-component reaction, to boost endosomal escape and mRNA delivery efficiency [25]. Their AID-lipid system supports applications like mRNA vaccines and gene-editing tools, offering a scalable and adaptable option for mRNA therapeutics. Additionally, Liu and colleagues developed a novel LNP system for organ-targeted mRNA therapy focused on the liver and lungs [26]. They found that cholesterol and phospholipids, though commonly used in LNP formulations, are not essential for effective delivery and instead contribute to unwanted liver accumulation. To overcome this limitation, they designed a three-component LNP system comprising nAcx-Cm lipids, permanently cationic lipids, and polyethylene glycol (PEG)-lipid. This formulation enables efficient pulmonary mRNA accumulation and translation, particularly in endothelial and epithelial cells. Compared to traditional four- or five-component LNPs containing cholesterol, the three-component system demonstrates superior efficacy, enhanced targeting specificity for lung delivery, and remarkable stability.

The further development of LNPs has led to the creation of cell-specific LNPs. Lian’s team demonstrated that incorporating stearic acid N-hydroxysuccinimide ester (SA-NHS) into LNPs enhanced bone marrow-homing (BM-homing) tropism, enabling transfection of 14 distinct cell types, including hematopoietic stem cells (HSCs) and immune cells [27]. The study highlighted the role of apolipoprotein E (ApoE) in BM-targeted delivery, potentially guiding new LNP designs for extrahepatic nucleic acid delivery in hematopoietic diseases. Li et al. explored the in situ engineering of CAR T cells using spleen-targeted LNPs, composed of DSPE-PEG2000-biotin conjugated with biotinylated anti-CD3 via streptavidin, to deliver CAR mRNA directly to T cells [28]. This approach simplified CAR T cell preparation and enhanced their anti-tumor function, showcasing the potential of organ-targeted mRNA therapies for solid tumors.

Despite their potential, LNPs face significant challenges in delivering ASOs. Byrnes et al. demonstrated that encapsulating ASOs in LNPs could enhance their activity by up to 100-fold in cultured primary brain cells compared to free ASOs. However, in vivo studies revealed a critical limitation: unlike free ASOs, LNP-delivered ASOs failed to achieve widespread mRNA downregulation across the brain following intracerebroventricular injection. This shortfall likely arises from ASO accumulation in cells lining the ventricles and blood vessels. Moreover, immune activation was observed post-dosing in a formulation-dependent manner, indicating that LNP encapsulation does not fully eliminate the cellular toxicities associated with the ASO backbone [72].

## 5. Extracellular Vesicles Delivery

Extracellular vesicles (EVs) have emerged as promising RNA delivery systems due to their natural ability to encapsulate and protect RNA molecules, enabling targeted delivery to specific cells [73]. Their biocompatibility and inherent targeting capabilities make them particularly attractive for therapeutic applications, especially in efficiently delivering RNA-based therapeutics [74].

Respiratory diseases, exacerbated by the COVID-19 pandemic, remain a significant global health challenge. While intramuscular mRNA-LNP vaccines have shown efficacy, their limited pulmonary bioavailability highlights the need for alternative delivery strategies. Inhaled nanoparticle therapeutics represent a promising solution, yet optimizing LNP formulations for efficient mRNA translation and pulmonary delivery remains a significant hurdle. In contrast, biological nanoparticles like EVs offer a compelling alternative, capitalizing on their natural ability to encapsulate mRNA and facilitate cellular delivery. Notably, EVs derived from pulmonary cells contain molecular components and membrane features uniquely suited to the lung microenvironment. For example, Popowski et al. demonstrated that lung-derived EVs (lung-Exo) exhibit superior distribution and retention of molecular drugs in deep lung tissues compared to synthetic nanoparticles. These lung-derived EVs thus represent a novel delivery system with enhanced pulmonary bioavailability, providing a tailored platform for delivering mRNA and protein-based therapeutics for lung diseases [29,30].

Beyond respiratory diseases, EV-based therapeutics have shown promise in other therapeutic areas. For instance, Ma et al. developed a clinically scalable method utilizing cellular nanoelectroporation with commercially available track-etched membranes (TM-nanoEP) to produce large quantities of small EVs (sEVs) loaded with therapeutic mRNAs and associated miRNAs. Unlike conventional methods that load therapeutics into EVs post-production, this approach uses parental cells as bioreactors. These cells endogenously transcribe plasmid DNAs (pDNAs), which generate therapeutic mRNAs that are subsequently packaged into multivesicular bodies (MVBs) or intraluminal vesicles (ILVs)—precursors of sEVs—and released as therapeutic sEVs (t-sEVs) via exocytosis. By optimizing electroporation parameters, TM-nanoEP achieved efficient loading of functional mRNAs (e.g., BMP-2 and VEGF-A) into abundant sEVs, significantly improving the efficacy of EV-based therapies. This method outperforms traditional approaches using naturally secreted or exogenously loaded RNAs like miRNAs and siRNAs. Moreover, the delivery of pDNAs not only enhances mRNA production but also promotes the secretion of miRNAs encoded by the pDNAs, creating a synergistic mRNA-miRNA therapeutic effect [31].

Recent advancements have further improved EVs by incorporating virus-derived structures, enhancing their potential for cancer and neurological disorder therapies. Wedge et al. explored EVs programmed by oncolytic viruses (OVs) to improve cancer treatment. These EVs, produced by virus-infected cancer cells, carry artificial miRNA (amiR-4), which targets and suppresses genes like ARID1A that contribute to cancer cell resistance against viral infections. This strategy allows EVs to spread amiR-4 to neighboring cancer cells, weakening their antiviral defenses and making them more vulnerable to viral attacks and small-molecule therapies. When combined with immunotherapies, this approach enhances cancer treatment by providing a broader, more targeted attack on cancer cells while minimizing harm to normal tissues [32]. Similarly, Gu’s team developed leukocyte-derived EVs containing retrovirus-like capsids to improve systemic mRNA delivery to neurons. This system effectively crosses the BBB, delivering mRNA in a neuroinflammation mouse model and offering an immune-inert platform for gene therapy in neurological disorders [33].

Further modifications to EV surface membranes have enhanced their utility in gene therapy for both cancer and neurological disorders. Cotto et al. developed a system for delivering mRNA to retinal photoreceptors by modifying EVs with cationic motifs. By anchoring cationic peptide carriers (CPC) and PEG groups to milk-derived EVs, they enhanced hydrophilicity and transport efficiency. This approach demonstrated excellent biocompatibility and successfully delivered mRNA to retinal cells, offering a promising method for localized gene therapy in retinal conditions [34]. In another study, Dong’s team engineered sEVs loaded with mRNA using microfluidic electroporation technology. By conjugating targeting ligands such as anti-CD71 and anti-PD-L1 antibodies to the sEVs, they achieved enhanced mRNA delivery to cancer cells, stimulating immune responses and offering a promising treatment for recurrent and drug-resistant tumors [35].

Although significant progress has been made in improving the targeting and delivery efficiency of EVs, loading large biomolecules into EVs remains a challenge. To address this, Malle’s team developed an innovative RNA-loading system based on a toehold-mediated strand displacement mechanism. The system facilitates the selective and efficient loading of mRNA into EVs by establishing a DNA-mediated fusion between mRNA-loaded liposomes and EVs. This method not only ensures efficient RNA loading but also allows for controlled RNA release and purification. The toehold structure plays a key role in precisely controlling RNA release from EVs, significantly enhancing delivery efficiency. Compared to conventional EV loading methods, this system improves RNA cargo selection and loading precision, thereby boosting the therapeutic potential of EVs for gene therapy and RNA-based treatments [75].

## 6. Other Nanomaterial-Based Delivery

Although RNA therapeutics have been successfully applied in the clinic using LNPs [76], challenges remain, particularly concerning toxicity. While cationic lipids in LNPs facilitate RNA encapsulation, they also carry toxicity risks [77]. For instance, cationic LNP components can interact with enzymes like protein kinase C, leading to cytotoxic effects [78,79]. High concentrations of these lipids can disrupt cellular membranes, causing cell lysis and necrosis [78,79]. To overcome these limitations, various nanoparticle carriers, including polymer-based (both natural and synthetic), inorganic, hybrid nanoparticles, and biological approaches using peptide nano-assemblies, have been actively studied [80].

### 6.1. Polymeric Nanoparticles

Naturally derived polymers, such as hyaluronic acid and chitosan, are widely utilized as mRNA carriers in drug delivery systems due to their biocompatibility and high modifiability. Among these, chitosan stands out for its cationic charge and ease of modification, which facilitate complex formation and surface adsorption [81]. Soliman et al. demonstrated that chitosan-based nanoparticles achieved a transfection efficiency of 60–65% in vitro by optimizing factors such as polymer length, degree of deacetylation, hyaluronic acid content, charge density, and nucleic acid composition [36]. Beyond mRNA delivery, chitosan nanoparticles have also been employed to deliver miRNA mimics, key biomarkers in cancer therapy [82]. These miRNA/chitosan complexes target macrophages to treat cardiovascular diseases, modulating gene expression both in vitro and in vivo. In mice, treatment with miRNA/chitosan nanoparticles reduced reverse cholesterol transport, highlighting their potential for addressing atherosclerotic lesions [83]. Furthermore, incorporating heparin into chitosan nanoparticles enhances transfection efficiency. Pilipenko et al. reported pH-dependent oligonucleotide release from heparin-loaded chitosan nanoparticles, driven by polyplex swelling and network collapse under mildly acidic conditions (pH 4.5). siRNA targeting vascular endothelial growth factor (VEGF) delivered via heparin-enriched chitosan nanoparticles showed 25% higher silencing efficiency in ARPE-19 cells compared to chitosan nanoparticles without heparin [37,84].

In addition to naturally derived polymers, synthetic polymers also play a critical role in RNA delivery. High-molecular-weight polyethylenimine (PEI) is considered one of the most effective non-viral cationic vectors for gene transfection. However, its high cytotoxicity limits clinical applications [85,86]. To mitigate this, fluorination was introduced to PEI, reducing cytotoxicity and enhancing its biodistribution control. Fluorinated PEI facilitated major accumulation of siRNA polyplex nanoparticles in the liver, while non-fluorinated PEI primarily delivered siRNA nanoparticles to the lungs following intravenous administration in mice [38]. Beyond cationic polymers, an anionic complex consisting of PEI, γ-polyglutamic acid, and mRNA demonstrated stable and high in vitro protein expression without cytotoxicity. Intravenous administration of this anionic complex resulted in high protein expression, predominantly in the liver and spleen [39].

### 6.2. Peptide-Derived Nanoparticles

Cell-penetrating peptides (CPPs) are short, cationic peptides (5–30 residues) capable of mediating intracellular delivery of diverse biological cargos, including large protein complexes and genetic materials, which typically struggle to penetrate cellular membranes. CPPs can traverse membranes via energy-dependent or independent mechanisms, making them promising candidates for peptide-based RNA delivery platforms [87,88].

While LNPs are widely used for systemic CRISPR-Cas9 delivery, their effectiveness is largely limited to the liver due to clearance mechanisms, restricting their application in extrahepatic targeting. In contrast, peptide-based nanoparticles offer advantages such as extrahepatic delivery, chemical diversity, and design flexibility [89]. Gonzalez et al. introduced a novel class of CPPs, termed “ADGN peptides”, which self-assemble into stable nanoparticles for efficient delivery of long mRNA and CRISPR components. These ADGN nanoparticles protect RNA, traverse cell membranes efficiently, and preferentially target cancer cells overexpressing the laminin receptor. In vitro, they achieved 60% CRISPR-Cas9-mediated knockout of the luciferase gene with a preference for G insertions at the target site. In vivo, systemically administered ADGN nanoparticles successfully delivered functional CRISPR-Cas9 systems to lung tumor cells, enabling effective gene knockout. Notably, their biodistribution depended on the peptide-to-RNA molar ratio, highlighting the tunability of this platform [40].

The KL4 peptide, modeled after pulmonary surfactant protein B (SP-B), has been utilized as an mRNA carrier with a linear PEG attachment. This PEG-KL4/mRNA complex exhibited superior transfection efficiency compared to naked mRNA or lipoplexes in mouse lungs after intratracheal administration [41]. Similarly, Levačić et al. developed a library of peptide-like oligoaminoamides with synthetic amino acids, histidine modifications, and bioreducible disulfide blocks, to enhance mRNA transfection efficiency [42].

### 6.3. Inorganic Nanoparticles

Inorganic materials possess ideal properties such as non-toxicity, hydrophilicity, biocompatibility, and high stability, making them invaluable in RNA packaging, protection, and delivery. Among these, gold nanoparticles (AuNPs) stand out due to their bioinert nature, ease of synthesis, and functionalization through thiol-gold bonds or electrostatic absorption. When functionalized with thiolated siRNAs, these nanoparticles—known as spherical nucleic acids (SNAs)—exhibit enhanced stability, high cellular uptake efficiency, and prolonged therapeutic activity. Notably, SNAs can cross the blood-brain and blood-tumor barriers to silence oncogenes and reduce tumor burden without inducing adverse side effects, thus paving the way for clinical applications [43]. Building on the SNA platform, gold cores modified with therapeutic agents or targeting ligands have been developed for gene and combination therapy. Alternatively, pristine RNAs can be noncovalently adsorbed onto polycation-capped AuNPs. For example, PEG-PEI-coated AuNPs have demonstrated potent siRNA delivery to pancreatic stellate cells, achieving effective silencing of HSP47 and reducing pancreatic cancer-associated desmoplasia [90].

Other widely applied bioinert carriers include carbon nanomaterials (e.g., carbon nanotubes, graphene, nanodiamonds) and mesoporous silica nanoparticles (MSNs). Functionalized carbon nanotubes deliver siRNAs to renal cells, halting acute kidney injury by silencing p53 and meprin-1b [44]. MSNs, with tunable pore sizes and cationic polymer coatings, enable RNA attachment and are ideal for combination therapy [45].

Dissolvable nanoparticles, such as calcium phosphate (CaP), reduce the risk of tissue accumulation and dissolve in acidic environments to release RNA cargo. Lipid-coated CaP nanoparticles significantly enhance systemic siRNA delivery to tumors [46]. Iron oxide nanoparticles, approved for imaging, serve as multifunctional platforms combining siRNA delivery, imaging, and magnetically controlled targeting for gene therapy [91]. Metal-organic frameworks (MOFs), with their high surface area, porosity, and biodegradability, offer efficient RNA delivery through coordination, encapsulation, or biomineralization, releasing RNA upon structural collapse and enhancing therapeutic efficacy [92].

## 7. Perspectives

Recent advancements in RNA delivery systems have shown great promise in therapeutic applications, particularly in vaccine development and disease treatment. Platforms such as viral vectors, LNPs, VLPs, and EVs each offer distinct advantages in improving the stability, specificity, and efficiency of RNA-based therapeutics (Table 1 and Table 2). Notably, LNPs have proven highly effective in the delivery of mRNA vaccines, as demonstrated by the success of COVID-19 vaccines, as well as in the development of siRNA-based drugs. However, each delivery system also presents unique challenges.

For instance, AAV have a limited DNA packaging capacity of 4.5 kb; recent studies indicate that AAV-delivered RNA molecules typically measure shorter than 2000 nt, although the maximum RNA length that AAVs can effectively accommodate is still unclear. Additionally, AAV’s transduction efficiency is dose-dependent: while higher doses can enhance efficiency, they also increase toxicity and immunogenicity [93,94,95].

In contrast, VLPs, which do not carry viral genomes, eliminate the risk of disease transmission. However, their virus-like symmetric and repetitive structures can provoke high immunogenicity. While this property makes VLPs promising vaccine candidates, it limits their use in therapeutic RNA drugs.

Furthermore, eukaryotic genomes contain domesticated genes from integrating viruses and mobile genetic elements, including homologs of the Gag protein found in LTR retrotransposons and retroviruses. Utilizing endogenous retrovirus-based delivery systems could potentially reduce immunogenicity, allowing for RNA delivery with minimal immune responses [21]. However, a major challenge remains: their transduction efficiency for targeted delivery is relatively low, highlighting the need for effective endogenous envelope glycoproteins to enhance targeting and delivery efficacy.

The toxicity of LNPs is particularly relevant in formulations containing cationic lipids, which are synthetic and whose toxicities may not be fully understood or have been observed in biological systems. In many cases, the effectiveness of a cationic lipid in LNP formulations is associated with increased toxicity. For instance, multivalent cationic lipids are more effective than monovalent ones in LNPs, but they also exhibit significantly higher toxicity to cells. Additionally, LNPs face significant challenges in delivering ASOs, as the delivered ASOs tend to accumulate in cells lining the ventricles and blood vessels.

To address the toxicity, various non-lipid nanoparticle carriers have been actively developed, including polymer-based, inorganic, hybrid nanoparticles, and biological approaches using peptide nano-assemblies. However, new challenges remain: polymeric vehicles suffer from low degradability and dose-limited toxicity with certain cationic polymers, as well as high charge density; the synthesis of inorganic nanoparticles is complex, limiting their scalability, and their low degradability may pose potential toxicity risks; peptide-derived nanoparticles often exhibit low stability and potential immunogenicity [92].

EVs have been explored as an endogenous alternative to LNPs, particularly in applications such as inhaled nanoparticle therapeutics. However, EVs present their own set of challenges, including heterogeneity, difficulties in large-scale production, and inconsistent RNA loading and targeting specificity—all of which must be addressed for successful clinical application [96].

The future of RNA delivery lies in combining the strengths of these systems to develop hybrid platforms that leverage their respective advantages. The study by Peng et al. effectively combines the advantages of LNPs and EVs by developing bio-functional lipid nanoparticles (LNP@HMVs) coated with neutrophil-bacterial hybrid cell membrane vesicles (HMVs). By integrating the targeting properties of EVs with the delivery efficiency of LNPs, this approach provides a promising strategy to combat antibiotic resistance and improve treatment outcomes in bacterial infections [97]. Zakas et al. explored a novel in vivo delivery approach based on an engineered transposase, Sleeping Beauty, delivered as an mRNA within a LNP, in combination with an rAAV-delivered transposable transgene [98]. Their findings offer the possibility of significantly reducing the dose used for clinical AAV gene editing therapies, promising to improve drug safety and reduce the cost of future gene therapies. Optimizing delivery systems through ancillary means can also be effective. Fitzgerald et al. demonstrated that using foam-based delivery carriers significantly improves transfection efficiency and homogeneity while reducing systemic exposure and targeting non-target tissues, thereby enhancing gene therapy safety [99]. Moreover, machine learning and AI are expected to play a critical role in optimizing nanoparticle design and improving targeting capabilities. Innovations in non-invasive delivery methods, such as ultrasound-based techniques, further contribute to enhancing the safety and efficiency of these therapies. A promising strategy to reduce immunogenicity involves utilizing endogenous retroviruses—rather than exogenous viruses—to construct fully endogenous delivery systems. This approach could facilitate RNA delivery with minimized immune responses. Additionally, certain endogenous retroviral envelope proteins, which are tissue-specific in their functions, can be leveraged as Env for endogenous VLPs to enhance the delivery and function of cargoed RNA. For instance, the primate-specific endogenous retroviral envelope protein ERVH48-1, which sequesters SFRP2 to regulate human cardiomyocyte development, could be used to improve RNA delivery in myocardial injury repair (Figure 1) [100].

Overall, as research progresses, RNA delivery systems will continue to evolve, making personalized medicine more achievable and providing novel treatments for previously untreatable conditions.

## Figures and Tables

**Table 1:** Comparative features of AAVs, VLPs, lipid nanoparticles (LNPs), and EVs as RNA delivery systems.

Name	Size	Genome	Advantages	Challenges
Adeno-Associated Virus (AAV) 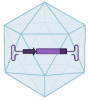	20–25 nm	Single-stranded DNA, up to 4.7 kb	High transduction efficiency in various cell types. Long-term expression in target cells. Low immunogenicity and safety profile.	Limited packaging capacity for larger genes. Pre-existing immunity in human populations can reduce effectiveness. Production and purification can be complex and costly.
Virus-Like Particles (VLPs) 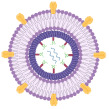	20–200 nm	No viral genome; can be engineered to carry RNA	Mimic natural viruses, enhancing cellular uptake and immune response. High stability and safety as they are non-infectious. Versatile for various types of genetic material.	Production can be challenging and require specific conditions. Limited capacity for carrying large RNA constructs. Potential immunogenicity depending on the source.
Lipid Nanoparticles (LNPs) 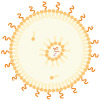	50–150 nm	Encapsulate mRNA or other RNA molecules	High delivery efficiency to a wide range of cell types. Protect RNA from degradation and facilitate endosomal escape. Scalable and adaptable for different RNA therapeutics.	Formulation can impact stability and delivery efficiency. Potential toxicity and immunogenicity issues. Optimization is required for organ-specific delivery.
Extracellular Vesicles (EVs) 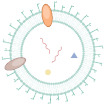	30–1000 nm	Naturally contain RNA and can load therapeutic RNA	Biocompatibility and low immunogenicity, as they are derived from cells. Natural targeting mechanisms can enhance delivery to specific tissues. Capable of delivering complex cargo, including proteins and nucleic acids.	Isolation and characterization can be complex and time-consuming. Variability in composition and efficacy based on source. Limited control over cargo loading and release mechanisms.

**Table 2:** RNA delivery systems and their targeting specificity for tissues and cell types.

Name	Modification	Target	Reference
Viral delivery	Replaced AAV’s inverted terminal repeats (ITRs) with RNA packaging signals (RPSs)	The blood-brain barrier (BBB)	Bai et al. [16]
	Developed circular RNAs with short-imperfect duplex regions (ds-cRNAs) containing ITRs for AAV packaging	Neurons and Microglia	Feng et al. [17]
	Designed microRNAs (miRNAs) targeting Atxn2, flanked by AAV2 145-bp ITR sequences, and packaged them into a peptide-modified AAV9 (PM-AAV9) vector	Central Nervous System (CNS)	Amado et al. [18]
Virus-like particle delivery	Used HIV-derived VLPs to develop an mRNA vaccine co-expressing HIV-1 Env and SIV Gag proteins	T cell	Zhang et al. [19]
	Engineered self-assembling VLPs by incorporating ESCRT proteins into the SARS-CoV-2 spike protein, enhancing spike protein enrichment on VLPs.	T cell	Hoffmann et al. [20]
	Developed selective endogenous eNcapsidation for cellular delivery (SEND) by engineering mouse and human PEG10 to package, secrete, and deliver specific RNAs.	Mammalian Cells	Segel et al. [21]
Lipid nanoparticle delivery	Synthesized a combinatorial library of ionizable, degradable lipids using reductive amination.	Lung	Jiang et al. [22]
	Developed charge-assisted stabilized lipid nanoparticles (CAS-LNPs) by optimizing surface charges with a peptide-lipid conjugate	Pulmonary	Liu et al. [23]
	Combined machine learning with combinatorial chemistry to identify the most effective candidates.	Muscle and immune cells	Li et al. [24]
	Develop a one-pot, tandem multi-component reaction based on the rationally designed amine–thiolacrylate conjugation	Lung and spleen	Han et al. [25]
	Designed a three-component LNP system comprising nAcx-Cm lipids, permanently cationic lipids, and polyethylene glycol (PEG)-lipid.	Lung	Su et al. [26]
	Designed a three-component LNP system comprising nAcx-Cm lipids, permanently cationic lipids, and lyethylene glycol (PEG)-lipid.	Liver and lungs	Lian et al. [27]
	Explored in situ engineering of CAR T cells using spleen-targeted LNPs, composed of DSPE-PEG2000-biotin conjugated with biotinylated anti-CD3 via streptavidin	T cells	Li et al. [28]
Extracellular vesicles delivery	Optimized exosomes by isolating them from lung tissues	Lung	Popowski et al. [29,30]
	Generated exosomes enriched with angiogenesis- and osteogenesis-related mRNAs (e.g., VEGF and BMP2) by engineering bone marrow mesenchymal stem cells (BMSCs)	Bone	Ma et al. [31]
	Explored EVs programmed by oncolytic viruses (OVs)	Cancer cells	Wedge et al. [32]
	Developed leukocyte-derived EVs containing retrovirus-like capsids	Neurons	Gu et al. [33]
	Anchoring cationic peptide carriers (CPC) and polyethylene glycol (PEG) groups to milk-derived EVs	Retinal photoreceptors	Cotto et al. [34]
	Engineered sEVs loaded with mRNA using microfluidic electroporation technology and by conjugating targeting ligands such as anti-CD71 and anti-PD-L1 antibodies to the sEVs	Tumors	Dong et al. [35]
Polymeric nanoparticles	Optimizing factors such as polymer length, degree of deacetylation, hyaluronic acid content, charge density, and nucleic acid composition	Macrophages	Soliman et al. [36]
	Incorporating heparin into chitosan nanoparticles	ARPE-19 cells	Pilipenko et al. [37]
	Fluorination was introduced to PEI	Liver	Xue et al. [38]
	An anionic complex consisting of PEI, γ-polyglutamic acid, and mRNA	Liver and spleen	Hamada et al. [39]
Peptide-derived nanoparticles	Introduced a novel class of CPPs by optimizing the sequence, which can self-assemble into stable nanoparticles and can be adjusted in concentration to determine their biodistribution	Lung tumor cells	Gonzalez et al. [40]
	The KL4 peptide, modeled after pulmonary surfactant protein B (SP-B), has been utilized as an mRNA carrier with a linear PEG attachment.	Lung	Qiu et al. [41]
	Developed a library of peptide-like oligoaminoamides with synthetic amino acids, histidine modifications, and bioreducible disulfide blocks	Tumor Cells	Krhač et al. [42]
Inorganic nanoparticles	Used spherical nucleic acids (SNAs) with a dense shell of RNA oligonucleotides conjugated to a nanoparticle core	Glioblastoma cells	Jensen et al. [43]
	Functionalized nanocarbon fibers were modified with targeting ligands for specific accumulation	Kidney cells	Alidori et al. [44]
	Mesoporous silica nanoparticles (MSNs) were designed for dual loading of chemotherapeutic drugs and siRNA, with surface functionalization to enhance endosomal escape and target delivery.	Breast cancer cells	Meng et al. [45]
	Lipid-coated biodegradable calcium phosphate nanoparticles (LCPs) were developed for systemic delivery, with optimized particle size and surface charge for tumor targeting.	Tumor cells	Li et al. [46]

**Table 3:** Comparison of retrovirus, lentivirus, HSV, adenovirus, and AAV for gene delivery.

	Tropism	Cloning Capacity	Genomic Integration	Duration of Expression In Vivo	Advantages	Disadvantages
**Retrovirus**	Dividing cells (hematopoietic cells).	Moderate (~7–8 kb).	Stable integration into the genome.	Long-term (due to stable integration).	Stable gene transfer in dividing cells.	Insertional mutagenesis, limited tropism.
**Lentivirus**	Broad (dividing and non-dividing cells).	Moderate (~7–8 kb).	Stable integration into the genome (both dividing and non-dividing cells).	Long-term (stable expression).	Stable expression in a wide range of cells.	Risk of insertional mutagenesis, immunogenicity.
**Herpes Simplex Virus (HSV)**	Primarily neurons, can be engineered for other tissues.	Large (~150 kb).	Episomal (no integration).	Long-term in neurons (latent potential).	High cloning capacity, long-term expression in neurons.	Latency and reactivation risks, immunogenicity.
**Adenovirus**	Broad (respiratory, liver, epithelial).	Moderate (~36 kb).	Episomal (no integration).	Short-term (due to immune response).	High transduction efficiency, large cargo capacity.	Short-lived expression, immune response.
**Adeno-Associated Virus (AAV)**	Limited but can be engineered (muscle, liver, retina).	Small (~4.0 kb).	Low-frequency integration.	Long-term (non-dividing cells).	Low immunogenicity, stable expression.	Small cloning capacity, pre-existing immunity.
